# Solution structure of recombinant Pvfp-5β reveals insights into mussel adhesion

**DOI:** 10.1038/s42003-022-03699-w

**Published:** 2022-07-25

**Authors:** Maria Agnese Morando, Francesca Venturella, Martina Sollazzo, Elisa Monaca, Raffaele Sabbatella, Valeria Vetri, Rosa Passantino, Annalisa Pastore, Caterina Alfano

**Affiliations:** 1 Structural Biology and Biophysics Unit, Fondazione Ri.MED, 90133 Palermo, Italy; 2 Department of Biological, Chemical and Pharmaceutical Sciences and Technologies (STEBICEF), University of Palermo, 90128 Palermo, Italy; 3 Department of Physics and Chemistry—Emilio Segrè (DiFC), University of Palermo, 90128 Palermo, Italy; 4 Biophysics Institute, National Research Council, 90143 Palermo, Italy; 5 European Synchrotron Radiation Facility, Ave des Martyrs, 38000 Grenoble, France

**Keywords:** Solution-state NMR, Translational research

## Abstract

Some marine organisms can resist to aqueous tidal environments and adhere tightly on wet surface. This behavior has raised increasing attention for potential applications in medicine, biomaterials, and tissue engineering. In mussels, adhesive forces to the rock are the resultant of proteinic fibrous formations called byssus. We present the solution structure of Pvfp-5β, one of the three byssal plaque proteins secreted by the Asian green mussel *Perna viridis*, and the component responsible for initiating interactions with the substrate. We demonstrate that Pvfp-5β has a stably folded structure in agreement with the presence in the sequence of two EGF motifs. The structure is highly rigid except for a few residues affected by slow local motions in the µs-ms time scale, and differs from the model calculated by artificial intelligence methods for the relative orientation of the EGF modules, which is something where computational methods still underperform. We also show that Pvfp-5β is able to coacervate even with no DOPA modification, giving thus insights both for understanding the adhesion mechanism of adhesive mussel proteins, and developing of biomaterials.

## Abstract (web summary)

Solution structure of byssal plaque protein Pvfp-5β secreted by the Asian green mussel *Perna viridis* gives molecular insight into mussel adhesion on wet surfaces.

## Introduction

How marine organisms, such as mussels, sea stars, and sandcastle worms, manage to adhere so firmly to wet surfaces such to resist to the strength of tides and stormy waves is a topic of increasing interest^1^. This is not only because of the importance of this property for naval industry, for which surface biofouling is a major concern, as it accelerates surface corrosion that requires costly surface maintenance. Even more interesting are the implications that a full understanding of such a tight adherence mechanism could have for the development of new biomaterials with properties that could be exploited in regenerative medicine, tissue engineering, and material science^2,3^.

Among the marine organisms with adhesion properties are mussels that have developed a strategy for strong underwater adhesion through the secretion of a protein-based stringy appendage called byssus. This consists of filaments formed by bundles of fibers inter-twinned together^4^. Each filament ends with a protein-rich plaque, containing the mussel foot proteins (mfps) characterized by adhesive features, that acts as a water-resistant glue and allows the fiber to firmly anchor to different substrates^5,6^. Chemically, the byssus composition consists of several different proteins that are all synthesized in the mussel foot, a large organ that in freshwater allows the mussel to pull through the substrate and move. Byssus is produced in a groove on the ventral surface of the foot and exuded as a viscous secretion, that gradually hardens and forms fibers upon the contact with water, in a coacervation process that has analogies with the formation of the amyloid fibers of Abeta peptides^7^.

Six mussel foot proteins have been identified in the most studied *Mytilus* genus (mfp-2, -3S, -3F, -4, -5, and -6)^8^. Proteins from this organism have weak adhesion energies (<5 mN/m) and the peculiarity to be biodegradable and thus environmentally friendly^9^, usually non-toxic and with low immune response properties^10^. A peculiarity of the mussel foot proteins is that they contain catecholic amino acid 3,4-dihydroxy-l-phenylalanine (DOPA), a derivative of tyrosine obtained by post-translational modification^11^. DOPA is known to bind to a wide variety of substrates through its ability to form hydrogen bonds, hydrophobic interactions, metal coordination, and covalent bonds^12–16^, and thus many studies focused so far on DOPA-derivated polymers for developing of new adhesive biomaterials.

In a previous study, we have characterized the nonDOPA-modified version of one of the mfp proteins from the Asian green mussel *Perna viridis* (Pvfp-5β)^17^. Among the proteins produced by this organism that also include Pvfp-3α and Pvfp-6, Pvfp-5β is first to be secreted and to establish interaction with the substrate making it a system of particular interest^18^. The sequence of this protein contains two tandem repeats with high homology with EGF modules and shares 47–50% identity with the EGF repeats of the Notch ligand Δ-like 1 protein^17,18^. EGF motifs are all-β proteins characterized by three conserved disulfide bridges, which may link calcium ions, and they have been observed also in other mussel-adhesive proteins, such as Mgfp-2 of *Mytilus galloprovincialis*^12,19,20^. We demonstrated that it is possible to produce recombinant Pvfp-5β in bacteria and that Pvfp-5β has low toxicity and intrinsic adhesive properties also in the absence of DOPA modifications^17^, comforting the possibility to use this protein as a surface-coating bio-material for medical applications including the regeneration of damaged tissues. These results have also been backed-up by other studies, which have demonstrated that DOPA-substituted residues are not necessary to produce strong wet-resistant adhesion, and DOPAminated proteins do not have higher adhesive properties than the corresponding non-DOPAminated versions^5,21,22^. Our results also align with recent studies that highlighted the importance of lysine residues (Lys) in mussel adhesion^23–26^. Indeed, both DOPA and tyrosine are prone to form cation–π bonds with flanking positively charged residues such as lysine, which is thought to increase the cohesive strength of adsorbed layers of mfps and induce a spontaneous liquid–liquid phase separation of a protein-rich fluid phase (complex coacervation) in salty solutions^27–29^.

More recently, another study showed that both the DOPA- modified and the non-modified Pvfp-5β are largely unstructured and that only the DOPA- modified Pvfp-5β exhibited liquid–liquid phase separation (LLPS) under seawater-like conditions, whereas the Tyr-only version forms only insoluble aggregates^30^. These results, at variance with our data, open two important questions: if the proteins were to be unstructured why has Nature used the EGF fold, which is well known to be stably folded also thanks to the disulfide bridges? Is DOPA the only paradigm for developing new mussel-inspired bioadhesives?

To explore these matters, we made another important step forwards the possibility of understanding the structural determinants of the adhesive properties of Pvfp-5β by solving its three-dimensional structure in solution and establishing that, despite the absence of a proper hydrophobic core, the structure in solution is relatively rigid with only local slow motions. We also prove experimentally that unmodified Pvfp-5β is able to undergo simple coacervation at neutral or basic pH, and picture the coacervation process caused by Pvfp-5β self-assembling by molecular docking.

Our results provide the foundations for gaining a better understanding of the structural determinants of the adhesion properties of mussel proteins.

## Results

### Pvfp-5β contains two tandem EGF modules

Our previously published 2D ^1^H–^15^N HSQC NMR spectrum of unmodified Pvfp-5β leaves little doubts that the protein is stably folded and monomeric, displaying peaks dispersion over a range of 9.7–6.7 ppm (Supplementary Fig. S1)^17^. We then decided to proceed with the determination of the three-dimensional NMR structure in solution of Pvfp-5β. Of note is that the HSQC spectrum of our recombinant Pvfp-5β is quite different from that recently published by another group that is consistent with a mostly unstructured protein^30^. The determination of the three-dimensional NMR structure in solution of Pvfp-5β was not an easy task despite the small size of the protein. This is because the NMR spectra assignment required a formidable effort due to the high content of tyrosines (20.5%), and prolines (9.6%). Nevertheless, virtually complete assignment could be achieved (Supplementary Fig. S1), and, except for Pro12, almost all the side chains of Pvfp-5β amino acid residues were successfully assigned. The assignment was submitted to the BMRB database under the accession number 51091.

Interestingly, we noticed that the chemical shifts of the twelve cysteines have all values compatible with their oxidized forms (Supplementary Table 1), at variance with our previous determination of the disulfide bridges by mass spectrometry, which had confirmed formation of only five of the six possible bridges^17^. We however noticed that a few resonances of residues along the whole chain have quite large deviations from the expected values. Thus, for precaution, we first ran ARIA calculations imposing only the five disulfide bonds found by MS: C13–C29, C31–C40, C45–C56, C50–C67 and C69–C78^17^. The resulted energetically best 20 structures had a backbone root-mean-square deviation (RMSD) of 1.43 +/– 0.39 Å considering all residues, and 1.17 +/– 0.25 Å considering only the residues involved in ordered tracts (3–4, 6, 8–34 36–38, 41–59, and 61–82). Eight violations of the distance restraints above 0.5 Å were noticeable in the final structural ensemble along with a poor overlap for traics 1–11 and 18–30 (Supplementary Fig. S2).

The overall structure of Pvfp-5β is in agreement with what expected from homology criteria, showing the presence of two tandem EGF modules formed by two solvent-exposed antiparallel β-sheets and random coil regions held together by the disulfide bridges (Supplementary Fig. S2). The first module contains only two of the three disulfide bridges expected for an EGF-like domain (C13–C29, C31–C40), while the second module contains three disulfide bridges as expected (C45–C56, C50–C67, and C69–C78). The remaining part of the structure is characterized by long random coil regions. These preliminary results confirm and extend previous predictions and prove beyond doubts that the protein is folded and structured.

### Dynamic studies provide experimental evidence of a quite rigid scaffold

**Fig. 1:**
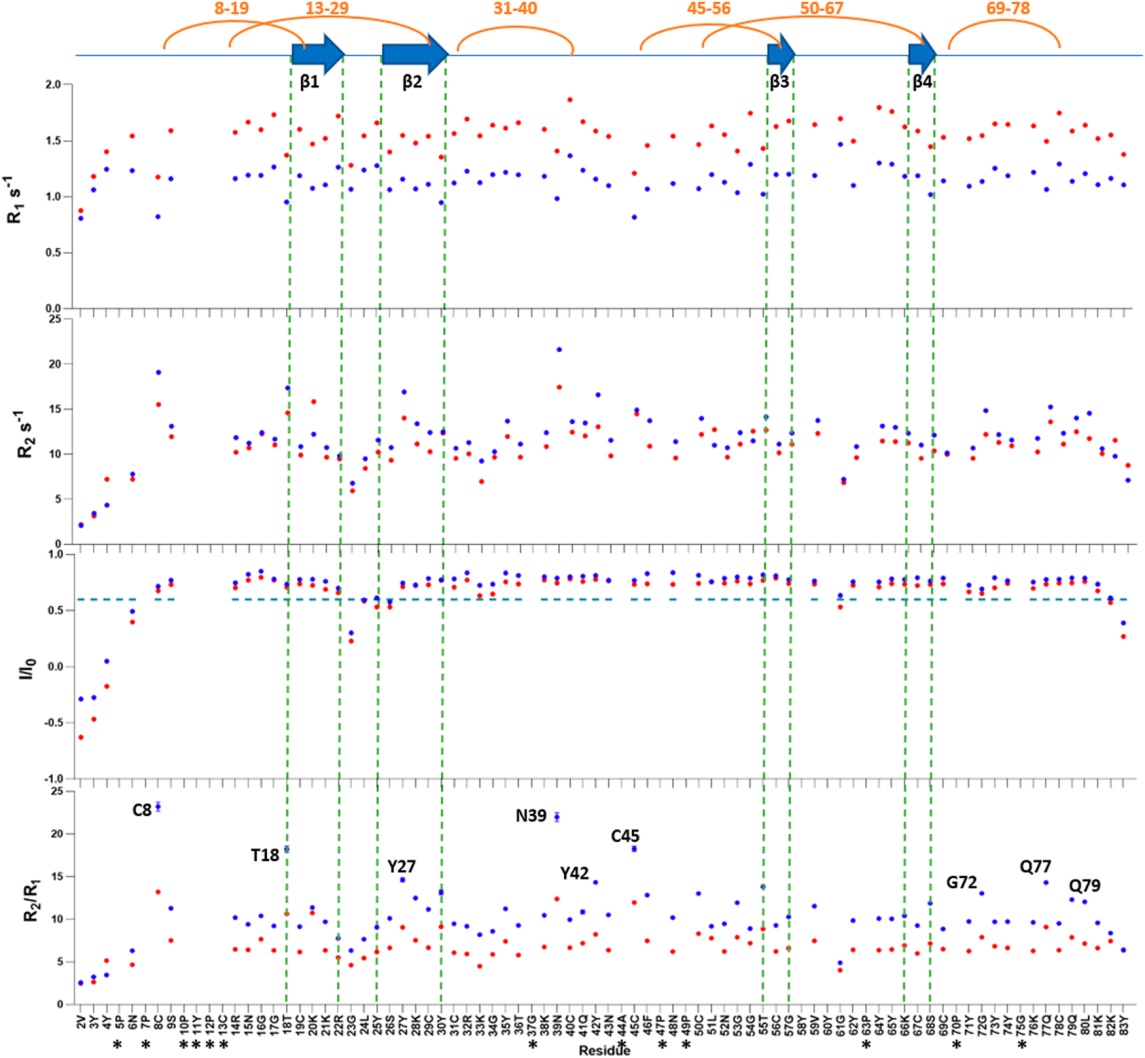
Dynamic features of Pvfp-5β. ^15^N-R_1_, ^15^N-R_2_, hetNOE, and *R*_2_/*R*_1_ ratio at 298 K measured for all Pvfp-5β residues at both 14.1 T (blue dots) and 18.8 T (red dots). β-strands are indicated as blue arrows at the top of the figure, while yellow curves indicate disulfide bonds. Black asterisks indicate proline residues or residues with peaks in overlap in the NMR experiments. In bold residues showing no-fast local motions within the ms-μs scale.

To investigate on the dynamics properties of the protein and concomitantly on the presence of the sixth disulfide bridge, we studied the protein relaxation at the ps-ns time scale. Despite Pvfp-5β contains only few secondary structure elements, the relaxation data indicate a relatively rigid protein, as revealed by the uniform values of ^15^N-R_1_, ^15^N-R_2_, and hetNOE, at both 14.1 and 18.8 T (Fig. 1). Elevated ^15^N-R_2_ together with low *R*_*1*_ and relatively high hetNOE values, were observed for residues C8, N39, Y42, C45, G72, Q77, and Q79, all belonging to unstructured regions and/or loops, and for residues T18 and Y27 belonging to β1 and β2, respectively. This indicates no fast-local motions within the μs-ms time scale for these residues.

To confirm that the observed dynamic features are intrinsic to the protein and independent from the experimental conditions, we repeated relaxation experiments at different temperatures (288, 293, and 303 K) at 18.8 T. The resulting hetNOE and *R*_*2*_*/R*_*1*_ ratios were plotted for each temperature, and showed no changes in the general trend (Supplementary Fig. S3). For a more reliable interpretation of the data, we also analyzed the data using the reduced spectral density map approach, which gives an unbiased qualitative picture of the protein motions^31^ (Supplementary Fig. S4). According to this analysis, Pvfp-5β exhibits a pattern typical of a rigid protein, with only residues 2–4 and 83 identifiable as flexible with little restriction of the backbone N–H vector motions. Residues C8 and T18 experience conformational exchange in the μs-ms time scale, supporting the presence of the sixth disulfide bridge between C8 and C19. We thus concluded that our previous MS results, which were relative to His-tagged Pvfp-5β, must have been affected by the presence of the N-terminal tag, which might have influenced the redox potential of the N-terminal C8 cysteine. Residues Y27, N39, G72, and Q77 also experience conformational exchange in the μs-ms time scale, while residues N6, G23 and G61 appeared in an intermediate situation between flexible and rigid, still experiencing fast motion moving towards low valued of *J*(0).

A correlation time of 7.8 ns was obtained from the T1/T2 ratios of 27 residues with T1 and T2 values within 1 standard deviation unit from the average. This value is slightly longer than what is expected for a monomeric globular protein of 9.5 KDa, but this is justified by the elongated ellipsoidal shape of Pvfp-5β, as confirmed by the ratio DII/DI (1.3) of the diffusion tensor derived from the orientation dependence of *R*_2_*/R*_1_, which indicates anisotropy^32^.

These observations provide invaluable information on the dynamics of the protein and lead us to conclude that Pvfp-5β is a monomeric protein at acidic pH, although it has a strong tendency to aggregate at neutral and basic pH.

### Refinement of the Pvfp-5β structure

**Fig. 2:**
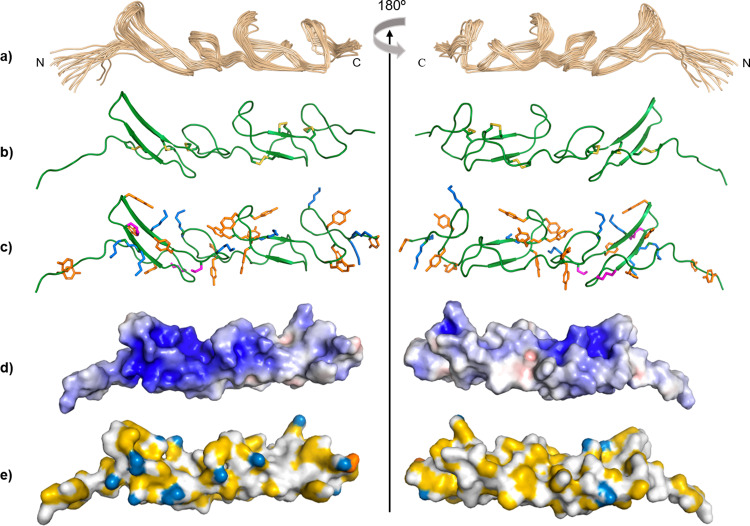
Solution NMR structure of Pvfp-5β. **a** Superimposition of the 20 lowest energy structures. **b** Cartoon representation of the structure at lowest energy with disulfide bridges in yellow. **c** Cartoon representation of the structure at lowest energy with tyrosines, lysines and arginines highlighted in orange, blue and magenta, respectively. **d** Electrostatic surface potential with acidic residues in red and basics in blues. **e** Hydrophobic surface.

A second structure calculation was performed imposing the extra disulfide bridge C8–C19 found by our dynamic studies. The energetically best 20 structures of Pvfp-5β have an RMSD of 1.42 +/– 0.5 Å for the backbone atoms of all residues, and of 1.09 +/– 0.29 Å only on the backbone atoms of the ordered residues 3–4, 6–82 (Fig. 2 and Table 1). The new structural ensemble resulted in a lower degree of variability in the long N-terminus in agreement with the relaxation data, disappearance of some consistent violations and an altogether better definition of the NMR bundle in agreement with the relaxation profile. The presence of the extra disulfide bond C8–C19 in the middle of an N-terminal random coil explains the ^15^N-R_2_ and hetNOE higher values and the presence of the exchange contribution at residue C8 as well as the partial order of the tract 9-16. We can thus consider this bundle as the structure representative of Pvfp-5β. The structure coordinates have been deposited in the Protein Data Bank with the accession code 7QAB.

**Table 1:** NMR and refinement statistics for the final 20 ensemble structures of Pvfp-5β.

**NMR distance and dihedral constraints**	
Distance constraints	
Total NOE	1696
Intra-residue	544
Inter-residue	1152
Sequential (|*i* – *j*| = 1)	621
Medium-range (|*i* – *j*| < 4)	164
Long-range (|*i* – *j*| > 5)	367
Intermolecular	0
Hydrogen bonds	9
Total dihedral angle restraints	112
*ϕ*	56
*ψ*	56
**Structure statistics**	
Violations (mean and s.d.)	
Distance constraints >1 Å	1
Dihedral angle constraints >5°	0
Deviations from idealized geometry	
Bond lengths (Å)	0,004 Å
Bond angles (°)	0,6 °
Impropers (°)	n/a
Average pairwise r.m.s. deviation (Å)	20 structures
Backbone of structured regions^a^	1.09 ± 0.29 Å
Heavy of structured regions	1.52 ± 0.37 Å
Backbone of all residues	1.42 ± 0.50 Å
Heavy of all residues	1.71 ± 0.49 Å

^a^Residues 3–4, and 6–82, selected on the basis of ^15^N backbone dynamics.

As in other EGF-like proteins, Pvfp-5β almost completely lacks a hydrophobic core, as also confirmed by our analysis of the temperature coefficients defined as the ratios dδ_HN_/dT detected over the range 283–303 K (Supplementary Fig. S5). When displaying the elongated molecule along the long axis, one of the two opposing surfaces is mainly positively charged (Fig. 2d). Mixtures of tyrosines and lysines are distributed along the whole protein (Fig. 2c). As expected, most of the residues located in unstructured regions and loops presented d*δ*_HN_/d*T* < –5 ppb/K, suggesting loose H-bonds^33^. Values of d*δ*_HN_/d*T* < –5 ppb/K were also obtained for residues K21, Y27, C56, and C67 belonging to both β-sheets and indicating exposure of these residues to the solvent. Residues T18, K20, R22, S26, K28, Y30, G57, Y64, Y65, K66, and S68, all in β-sheet regions, presented d*δ*_HN_/d*T* > –5 ppb/K confirming their involvement in secondary structure elements and stable H-bonds. Values of d*δ*_HN_/d*T* > –5 were also observed for few residues located in U-turns between β-sheets, in the loops, and in both N- and C- tails in agreement with an overall relatively rigid structure (Supplementary Fig. S5).

As a mean of structure validation, we modeled Pvfp-5β by Artificial Intelligence (AI) using the RoseTTAFold software in the Robetta server^34,35^. We obtained five models with high values of confidence (0.92) and with all the six disulfide bridges. Comparison of these models with the final experimental structural ensemble shows only minor differences interesting the interface between the two EGF-like modules, which is slightly different due to a difference in the loop β3-β4 (Supplementary Fig. S6). However, the NMR experimental structure is directly supported by unambiguous NOEs between the two repeats, and gives a degree of details that can hardly be expected from the very nature of machine learning techniques. Despite the enormous capability of artificial intelligence to predict structure, there might be details that still remain above prediction.

### Pvfp-5β is able to coacervate and undergo LLPS also without DOPA modifications

In a previous study, we demonstrated by dynamic light scattering (DLS) that Pvfp-5β has propensity to aggregate under conditions of pH shock^17^. In the present work, we analyzed the self-assembly process of Pvfp-5β by ThioflavinT (ThT) fluorescent dye, which is sensitive to the formation of intermolecular β structures in amyloid fibrils^36^ and accumulates in coacervates^37,38^. ThT fluorescence was monitored both by confocal microscopy and fluorescence lifetime imaging microscopy (FLIM) to detect the different species formed after the exposure of Pvfp-5β monomers to different pHs.

The exposure of Pvfp-5β to an alkaline environment (0.1 M Tris-HCl pH 8, 1 M NaCl) produces the progressive appearance of three main protein species. A sudden appearance of droplets of coacervation with diameter of about 1 μm or less is observed soon after the insult (Supplementary Fig. S7a, Supplementary Movie). Monitoring the sample over 2 h, reveals the formation of progressively larger ThT-positive species with a final appearance of fibrillar structures (Supplementary Fig. S7b, c). As a control, incubation of Pvfp-5β to pH 4.5 does not result in any ThT-positive species (Supplementary Fig. S7d).

**Fig. 3:**
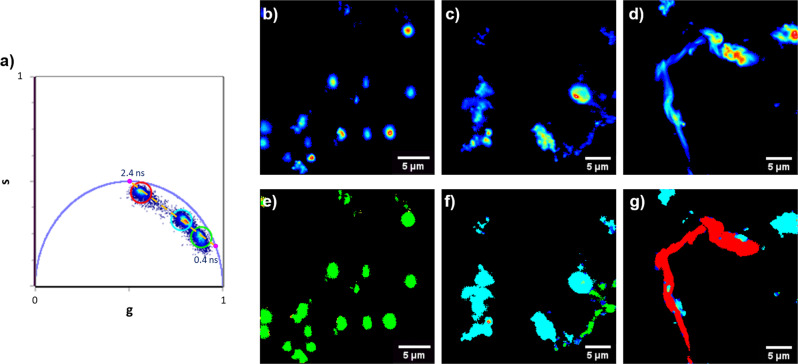
Coacervation of Pvfp-5β in alkaline environment. Phasor analysis of 256 × 256 pixels FLIM measurements on ThT signal of 1 mg/ml Pvfp-5β in 0.1 M Tris-HCl pH 8, 1 M NaCl. **a** Phasor plot obtained from images of the different species present in the sample. The coordinates g and s correspond to sine and 30 cosine transforms of each point in the phasor plot, respectively. Three distinguishable lifetime distributions, highlighted by colored circular cursors, are evident and lay on a straight line (dashed yellow) connecting two mono-exponential decays phasors with characteristic lifetime of 2.4 ns and 0.4 ns. **b**, **c**, **d** Fluorescence intensity images, from low intensity (blue) to high intensity (red) of coacervate droplets, medium size aggregates and fibril, respectively. **e**, **f**, **g** Lifetime maps: each pixel is colored according to the corresponding color code of the cursor highlighting the lifetime distributions in the phasor plot a).

To clarify the structural architecture of the protein assemblies at the submicron scale and probe the evolution of microscopic structural changes, FLIM was used to analyze the ThT fluorescence lifetime^39^. This has a specific sensitivity to environmental polarity, presence of specific residues (e.g., aromatics), or spacing between β-strands^40^. In aqueous environment ThT has a lifetime in the picosecond range, while longer lifetimes are measured in media with higher viscosity^41^. FLIM measurements were analyzed using the phasor approach, detailed in the Methods section, that provides a global view of the decay of fluorescent molecules, not imposing any specific model as required by fitting procedures (Fig. 3)^42,43^. Measurements on ThT-stained Pvfp-5β in alkaline environment reveal three different ThT fluorescence lifetime distributions, identified as distinguishable clouds of points in the phasor plot (Fig. 3a), each corresponding to one of the three main ThT-positive species (droplets of coacervates, medium size assemblies and fibrillar species). Each ThT lifetime in Pvfp-5β sample is characterized by a double exponential decay, whose main components have characteristic lifetimes of *τ*_1_ = 0.4 ns and *τ*_2_ = 2.4 ns, previously found for ThT-stained amyloid structures (Fig. 3a)^44^. The fastest decay was attributed to less-specific binding sites. In these cases, ThT fluorescence occurs because of increased environmental viscosity. Slower decays were attributed to more-specific interaction between ThT and intermolecular β-structures that add more constraints and less flexibility to the ThT-binding site. Shorter lifetimes were measured for the readily formed micronscale spherical protein condensates revealing their nature of droplets of coacervation (Fig. 3d). The ThT lifetime progressively increases for larger species, resulting longer for structures with fibrillar morphology, indicating packed/dense intermolecular β-structures (Fig. 3e, f). These results clearly demonstrate that non-modified Pvfp-5β exhibites LLPS under seawater-like conditions, at variance from a recent study that shows that only the DOPA-modified Pvfp-5β exhibited LLPS, whereas the non-modified version forms only insoluble aggregates^30^.

### Predicting the surface of interaction

**Fig. 4:**
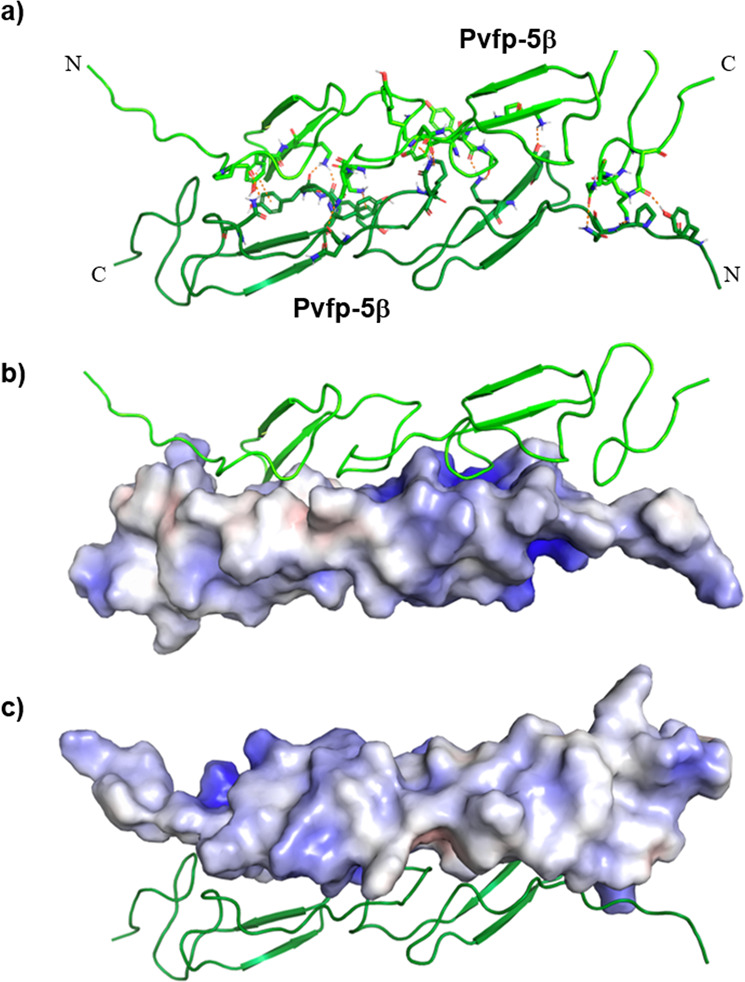
HADDOCK model of Pvfp-5β dimer. Lowest energy structure of Pvfp-5β/Pvfp-5β dimer as calculated by HADDOCK. **a** cartoon representation with residues of the two components involved in the interaction showed in sticks. **b**, **c** electrostatic surface potential for one monomer and cartoon representation for the other one.

To gain a pictorial impression of the Pvfp-5β coacervation process occurring in mussel adhesion, we used the experimental structure of Pvfp-5β to perform molecular docking of the Pvfp-5β/Pvfp-5β dimer to predict, which surface could initiate self-assembly. Docking of the dimer by HADDOCK resulted in three clusters, among which the first one is the most populated (177 structures) with the best HADDOCK and *Z*-score with a total RMSD of 0.6 ± 0.3 Å from the overall lower-energy structure (Supplementary Table 2). The resulting dimers are placed head-to-tail with a *D*_*2*_ axis of symmetry with a well-structured hydrophobic core involving Y27, Y62, and Y65 from both monomers (Fig. 4). The complex is stabilized by a number of π-π and cation-π interactions and by an extended net of hydrogen bonds in the interface between the two monomers (Supplementary Table 3). Further docking between the dimers proposes that they could interact with each other through additional exposed tyrosines on the external surface of the dimer. This would imply an orientation of the elongated monomer structure perpendicular to the fiber axis.

## Discussion

In this work, we report the experimental structure in solution of Pvfp-5β, one of the mussel proteins involved in surface adhesion through formation of the byssus plaque. The protein sequence contains two tandem EGF modules connected by a linker. EGF-like modules are evolutionary conserved motifs characterized by three conserved disulfide bridges, which hold together a structure otherwise too small to contain a proper hydrophobic core. Because of the presence of the disulfides, they are usually stably folded and are found in the extracellular domains of membrane-bound proteins and in proteins known to be secreted. It is thus reasonable to assume that they will also lead to a structured protein in Pvfp-5β.

Accordingly, our work confirms these expectances and provides more insights. First of all, it needs to be clarified that there is some confusion in the literature where Pvfp-5β is declared as a random coil protein while at the same time it is recognized to contain EGF-like motifs^17,18,30^. It is true that Pvfp-5β has long unstructured loops, but, at the same time, it is utterly incorrect to consider EGF as an intrinsically unstructured protein: as all small modules (less than ca. 50 amino acid), it needs disulfide bridges to be held in place, as it is seen in several neurotoxins and in BPTI^45^. Accordingly, the features of the CD spectrum are not those of a random coil since the single minimum is shifted to 205 nm (to be compared to 200 nm in random coil proteins) and strongly resembles that of another small but well-structured protein module, the WW domain^46^, which also shares with Pvfp-5β a positive band around 230 nm that is likely caused by the stacking of aromatic side chains^17^. In agreement with this view, we observed a whole network of intramolecular NOEs, which reassured us that the protein is folded and has a robust structure even in the absence of a proper hydrophobic core. We also have conclusive direct evidence that, under acidic conditions, Pvfp-5β is monomeric.

Our results are thus at strong variance with a recent paper in which NMR spectra are reported both for tyrosinated and DOPAminated Pvfp-5β^30^. In this paper, the authors showed one- and two-dimensional spectra, which have all the features of an aggregated fully unstructured protein. The same authors were unable to observe NOEs, which we instead observe extensively, as it is expected for a monomeric EGF-like protein. The discrepancy is of course possible given that Pvfp-5β is a protein difficult to produce, especially in bacteria, where it forms inclusion bodies that require to be solubilized by a robust and reliable protocol of refolding.

It has been independently suggested that the rigidity of the Pvfp-5β fold is needed to promote adhesion and byssus formation. Indeed, our NMR relaxation parameters, such as hetNOEs, clearly allow us to state that the backbone is relatively rigid with increased flexibility in the ps-ns time scale observed only at the termini and in loop β1/β2. Further spectral density map analysis allowed us to identify residues that seemed affected by μs-ms time scale motion, among which N39, in the long disordered loop between β2 and β3, is the most affected. It is therefore reasonable to consider that N39 acts as a hinge among the two EGF modules. Both lysine and tyrosine residues appear exposed to the solvent and can thus act synergically to interact with surfaces also in the absence of DOPA modifications. The overall rigidity, favored by the presence of disulfide bridges, could thus be a main structural feature to perform function through favoring the persistent exposure of tyrosine and lysine residues to the solvent, maximizing the interaction with surfaces and/or other proteins, while minimizing the entropy penalty.

**Fig. 5:**
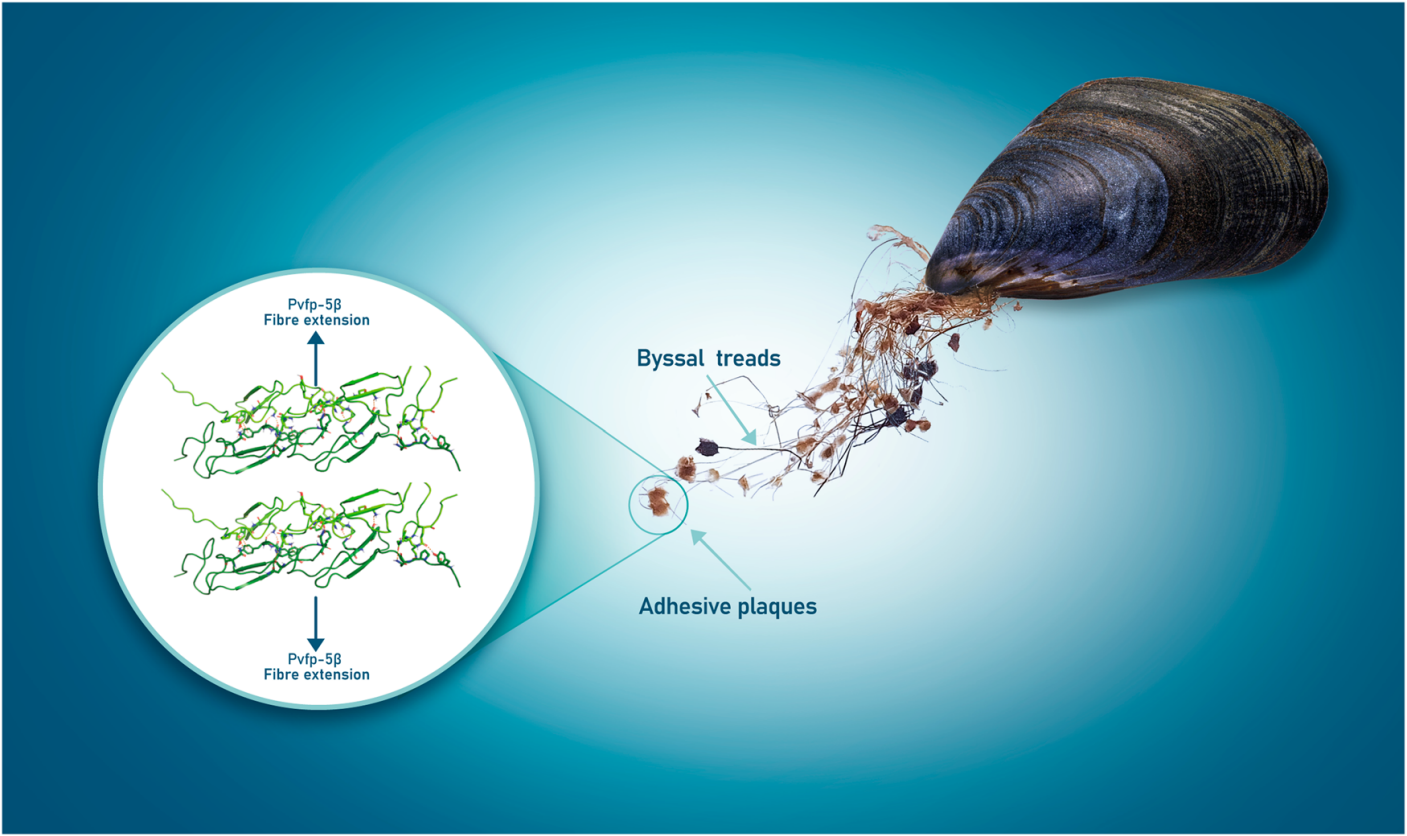
Pvfp-5β self-assembly in mussel byssal plaques. The elongated shape of the Pvfp-5β and the exposure of many interaction hotspots are ideal for fiber formation. Different units might pile up interacting in a way perpendicular to the main axis of the fibers, as in the cross-β of an amyloid fiber.

The fold and residues distribution also explain why Pvfp-5β is the frontline protein in the process of adhesion in *Perna viridis*. Its elongated shape and the exposure of so many interaction hotspots are ideal for adhesion to marine substrates. Based on the structure, it is thus easy to predict that, in the byssus plaque, the different monomers will pile up interacting in a way perpendicular to the main axis of the fibers, as in the cross-β of an amyloid fiber (Fig. 5). When considering the other byssal plaque components, we could reasonably assume that the complex will expose part of the cleft formed by the unstructured linker between the two EGF modules. This cleft could accommodate other foot proteins that could contribute to the fiber formation.

Finally, we demonstrated that DOPA is not required also for LLPS as we observe coacervation simply by changing the pH and salt concentration of the solution. The different behavior observed in a previous study^30^ could again be explained by the different nature of the samples. This is in full agreement with our results and others’ results showing that DOPA is not essential for adhesion^17,22^.

In conclusion, our work constitutes a direct structural attempt to understand the molecular recognition of mussel proteins at the molecular level and provides a model for mussel byssal plaque formation. Our results may explain why DOPA can be important for Pvfp-5β coacervation but does not contribute to the adhesion properties of this protein in agreement with experimental evidence^17,22^. DOPA can easily favor packing and more effective cross-linking of the monomers in the coacervate and contribute to its stability. Several questions remain open on the coacervation process in mussel adhesion: we do not know, for instance, the complex stoichiometry or the relative contribution of the different components, nor the precise kinetics of events that may take place. We also do not know precisely how the presence of DOPA could influence the binding mode. More work will thus be necessary to address these important open questions.

## Methods

### Protein production

Cloning, bacterial expression in *E. coli* and purification were achieved as previously published with minor adjustments^17^. Briefly, recombinant Pvfp-5β was expressed in BL21(DE3)pLysS *E.coli* cells as a TEV protease cleavable N-terminal His-tagged protein. Expression was induced by 1 mM Isopropil-β-d-1-tiogalattopiranoside for 3 h at 37 °C. Labeling was achieved by growing the cells in minimal medium using ^15^N ammonium sulfate and ^13^C glucose as sole nitrogen and carbon sources. The cells were disrupted by ultrasonic homogenizers. The inclusion bodies were re-solubilized in 8 M urea, 1 M NaCl, 2 mM DTT, 20 mM sodium phosphate buffer at pH 7.4. The supernatant was passed throw a 5-ml HisTrap FF crude column prepacked with Ni-Sepharose (GE Healthcare Life Sciences). The protein was eluted under denaturing and reducing conditions with a linear gradient of imidazole. Refolding was then performed by extensive dialysis at 4 °C, first in 20 mM sodium phosphate (pH 7.4), 2 M urea, 250 mM NaCl, 2 mM reduced gluthatione (GSH), and 0.5 mM oxidized gluthatione (GSSG), then in the same buffer with no urea, and finally in 20 mM sodium phosphate at pH 7.4, and 250 mM NaCl. His-tag removal was performed by adding TEV protease at a 1:50 molar ratio to the protein solution, and incubating at room temperature for 2 h. Cleaved Pvfp-5β was recovered by reverse IMAC chromatography. Because of its strong adhesive features, cleaved Pvfp-5β was not collected in the column flow through as expected, but it was eluted with a step gradient of imidazole, which allowed its separation from the tag. Extensive dialysis was then performed at 4 °C in 20 mM sodium phosphate at pH 7.4, and 250 mM NaCl to remove imidazole, and then in 5% acetic acid, which allowed easy lyophilization of the protein sample. Protein purity was verified by SDS-PAGE analysis. Protein concentration was assessed by UV spectrophotometric determination at 280 nm (extinction coefficient [ε] = 26080 M^−1^ cm^−1^).

### NMR measurements and spectral assignment

Three-dimensional NMR measurements for resonances assignment were performed at 800 MHz on Bruker spectrometers and 298 K. T1, T2, and heteronuclear NOEs were measured both at 600 and 800 MHz on Bruker spectrometers and 298 K using standard pulse sequences. Two different samples were prepared in 20 mM Acetate buffer pH 4.5, one at a concentration of ~350 μM, the second at ~900 μM. All NMR spectra were processed using nmrPipe^47^ and analyzed by CCpnmr software^48^.

^15^N,^1^H HSQC^49^ peaks allowed to identify the resonances of the backbone amide groups that were then assigned to the corresponding amino acids by analysis of HN(CO)CACB^50^, HN(CO)CA^51^ and HNCA^52^, HNCACB^53^ experiments, which also provided chemical shift values of Cα and Cβ atoms leading to sequence-specific assignment. The HNCO^54^ spectrum was used to assign the carbonyl carbon chemical shifts, while chemical shift values of Hα and Hβ were assigned by HBHA(CO)NH^55^ and HBHANH^56^ experiments. HN, and N resonances of 73 out of 75 non-proline residues were assigned and were used in the TALOS + software^57^, together with assigned resonances of HA, CA, CB, and CO, for empirical prediction of dihedral angles *ψ* and *ϕ*, and secondary structure.

Side-chain assignment was performed by 3D HCCH-TOCSY^58^ experiments, individually recorded for the aliphatic and aromatic chains. 2D (HB)CB(CGCD)HD^59^ and (HB)CB(CGCD)CEHE^60^ spectra were used to assign the Hδ and Hε protons of aromatic residues. Missing resonances were assigned by 13C NOESY-HSQC^61^.

### NMR structure calculation

The structure of Pvfp-5β was calculated with ARIA2.3^62^. Input data were the Pvfp-5β amino acid sequence, the chemical shift assignment list, the NOE restraints’ lists, dihedral angles obtained by TALOS+, and hydrogen bonds obtained by Rosetta protein modeling suite^63^. Disulfide bridge constraints were also imposed on the C13–C29, C31–C40, C45–C56, C50–C67, and C69–C78 pairs according to previous mass spectrometry analysis^17^. Additional structure calculation was performed imposing an extra disulfide bond involving C8 and C19 as suggested by relaxation measurements. NOE restraints were assigned manually by analysis of 3D ^15^N NOESY-HSQC^64^, 3D ^13^C NOESY-HSQC^61^ centered on the aliphatic region (0–6 ppm), and 3D ^13^C NOESY-HSQC centered on the aromatic region (6–8 ppm). Ambiguous NOE assignments were sorted automatically by ARIA throughout eight iterations, selecting the best 100 conformers at each iteration. A different violation threshold was set for each iteration: 200.0 for it0, 6.0 for it1, 3.0 for it2, 2.0 for it3, 1.0 for it4 and it5, 0.5 from it6 to it8. The conformers with the lowest energy values were used to filter the distance restraints from false positives and assign ambiguities. ARIA calculations were performed with the adaptive choice of violation tolerance. The assignment tolerance on the NOESY peak lists frequencies was 0.02 ppm for ^1^H, and 0.4 ppm for both ^15^N and ^13^C nuclei. Hydrogen bond restraints were imposed for the pairs T18-Y30, K20-K28, T55-S68, and G57-Y66. A log-harmonic distance restraint potential was assumed. This potential derives from a Bayesian analysis showing that NOEs and the derived distances ideally follow the log-normal distribution^65,66^. A log-harmonic potential was applied during the second cooling stage of the simulated annealing and during water refinement. The structural ensemble was visualized and analyzed using Chimera and Pymol^67,68^. Quality validation was performed using PROCHECK^69^ and the Protein Structure Validation Software suite (PSVS, https://www.bio.tools/psvs#!).

### NMR thermal coefficients measurements

Amide ^1^HN chemical shift temperature coefficients of Pvfp-5β were determined by recording a series of two-dimensional ^15^N,^1^H HSQC spectra at 283, 288, 293, 298, and 303 K, using a Bruker spectrometer operating at 800.03 MHz proton frequency. All spectra were referenced to the water signal for each temperature, processed using nmrPipe^47^, and analyzed by CCpnmr software^48^. Water chemical shift was referenced to 3-(trimethylsilyl)propane-1-sulfonic acid (DSS), which has negligible temperature dependence and allows analysis unbiased by deuterium lock artefact. The chemical shift values *δ*_HN_ of all residues were plotted as a function of the temperature. The data were analyzed assuming that residues with d*δ*_HN_/d*T* < –5 ppb/K form weaker H-bonds and should be considered secondary structure breakpoints or, if in an unstructured region, as promoters of H-bonds with water. Values of d*δ*HN/d*T* > –5 ppb/K correspond to the formation of tighter bonds likely involved in secondary structure elements^70^.

### Relaxation experiments

Backbone dynamics of Pvfp-5β were probed at 298 K with the ^15^N-R_1_, ^15^N-R_2_ relaxation and {^1^H}–^15^N heteronuclear nuclear Overhauser effect (hetNOE)^71–74^ under two different magnetic fields (14.1 and 18.8 T). ^15^N-R_1_, ^15^N-R_2_ and {^1^H}–^15^N NOE were also measured at other three temperatures (288, 293, and 303 K) under the solely magnetic field of 18.8 T. In all cases, *R*_1_ and *R*_2_ were measured with delays varying from 0.01 to 2 s, and between 0.017 and 2.7 s, respectively. {^1^H}-^15^N steady-state NOE data were obtained by measuring two spectra: an initial spectrum recorded without the initial proton saturation and a second spectrum recorded with an initial proton saturation of 8 s. The NOE values were then determined from the ratios of the average intensities of the peaks with and without proton saturation.

The relaxation data acquired at 298 K, obtained at both 18.8 T and 14.1 T magnetic fields, were analyzed by standard mathematical Modelfree formalism^75^ available in Dynamic Center tool provided by Bruker topspin platform. The experimental data were processed and fitted using two different structures with 5 and 6 disulfide bridges. Further analysis of the relaxation data acquired at different temperatures was carried out using the spectral density function approach^31,32^. The temperature was checked by monitoring chemical shift changes of selected peaks. No sample overheating was observed as a result of applying radiofrequency pulses. Out of the 83 amino acids present, fifteen residues were excluded from the analysis either because of being prolines or because of overlapping in the ^15^N HSQC (P5, P7, P10, Y11, P12, C13, G37, A44, P47, P49, Y58, Y60, P63, P70, G75).

### Structure predictions by artificial intelligence methods

Structural models of Pvfp-5β were obtained using the RoseTTafold suite^76^. This is one of the computational tools based on deep learning network able to predict protein structure from its amino acid sequence. The deep learning network is able to perform a series of 1D, 2D, and 3D convolution operations to increase the accuracy of the resulting protein structural model. The combinations of multiple sequence alignments, distance maps and three-dimensional coordinates representations give rise to better structural model in comparison to the more traditional prediction methods based on multiple sequence alignments and contact maps. The software generated five models, whose differences are highlighted as angstrom error estimates versus the position of each residue in the sequence. The goodness of the prediction is defined by a level of confidence, that is related to predicted lDDT (local distance difference test) using in DeepAccNet^77^. We obtained a level of confidence of 0.92 for Pvfp-5β.

### Confocal microscopy

Lyophilized Pvfp-5β was dissolved in 10 mM acetic acid up to the final concentration of 10 mg/ml. The solution was then diluted to 1 mg/ml in 20 mM sodium acetate at pH 4.5 or 0.1 M Tris-HCl at pH 8, and 1 M NaCl, and stained with 40 µM of an aqueous solution of ThT. 250 microliters of stained samples were placed on microscope chambered slides and imaged at 1024 × 1024 pixels resolution using a Leica TCS SP5 confocal laser scanning microscope, using 63x oil objective (Leica Microsystems, Germany). Leica white light laser was set at 470 nm excitation and ThT emission was detected in the range 485–585 nm.

### Fluorescence lifetime imaging microscopy

FLIM data were acquired in the time domain by means of a picoHarp 300 standalone TCSPC module (Picoquant), using a 63× oil objective (Leica Microsystems). 256 × 256 pixels images were acquired at a scanning frequency of 200 Hz, with Leica white light laser set in order to excite ThT (*λ*_ex_: 470 nm, *λ*_em_: 485–585 nm). FLIM calibration of the system was performed by measuring the known lifetime of fluorescein that is a single lifetime of 4.0 ns. FLIM data were analyzed by the phasor approach^42,43^ by the SimFCS software developed at the Laboratory of Fluorescence Dynamics, University of California at Irvine (www.lfd.uci.edu). The phasor approach is a Fourier domain technique that allows graphical analysis of FLIM measurements. It transforms the fluorescence decay measured in each pixel of the image to a single point called “phasor” in a polar representation. All possible single-exponential decays lay on a semicircle (defined as universal circle), with radius 1/2, going from point (0, 0), corresponding to *τ* = ∞, to point (1, 0), corresponding to *τ* = 0. Complex decays are linear combination of single-exponentials and are represented within the semicircle. Given that the phasors follow vector algebra, it is possible to geometrically resolve the fractions of two fluorescent species (in the simplest case) by the lever rule of vector additions. The linear combination of two single-exponential decay components generates phasors within the universal circle, which lay on a straight line joining the phasors of the two single components. The contribution/fraction from one single component to the lifetime is proportional to the distance of the phasor from it.

For Pvfp-5β measurements, the lifetime distributions were selected with green, cyan and red cursor and corresponding pixels were localized with the same color code in the images. Green pixels are related to shorter lifetimes. The progressively increasing lifetimes are mapped using cyan and red color.

### HADDOCK calculation

Docking calculations of protein–protein interactions for the Pvfp-5β/Pvfp-5β complex were performed ab-initio using the integrative modeling platform HADDOCK2.4^78,79^. During the HADDOCK docking protocol, the interacting partners are treated as rigid bodies in the initial stage, while the second stage introduces flexibility to the interacting partners through a three-step molecular dynamics-based refinement in order to optimize interface packing. Residues belonging to the interface region are then allowed to move their side-chains in a second refinement step. A final refinement in a solvent shell is then performed to improve the energetics of the interaction. The resulting protein–protein complexes are ranked in function of the HADDOCK score, which is a weighted sum of several terms such as electrostatic, van der Waals, and distance restraints energies, and buried surface area.

### Reporting summary

Further information on research design is available in the Nature Research Reporting Summary linked to this article.

## Supplementary information

Supplementary Information

Description of Additional Supplementary Files

Supplementary Movie

Supplementary Data

Reporting Summary

## Data Availability

The NMR assignment was submitted to the BioMagResBank (BMRB: www.bmrb.wisc.edu, 10.1093/nar/gkm957) under the accession number 51091. The NMR structure coordinates have been deposited in the Protein Data Bank (PDB: www.rcsb.org, 10.1007/978-1-4939-7000-1_26) under the accession code 7QAB. All other data are available from the corresponding author on reasonable request.
