# Supplementary material for: Solution structure of recombinant Pvfp-5β reveals insights into mussel adhesion
Source: Commun Biol. 2022 Jul 25;5:739. doi: 10.1038/s42003-022-03699-w (PMC9314366; doi:10.1038/s42003-022-03699-w)
Supplement: Supplementary file 1 — Supplementary Information [file 42003_2022_3699_MOESM1_ESM.pdf]

SUPPLEMENTARY INFORMATION

Maria Agnese Morando[1ǂ], Francesca Venturella[1ǂ], Martina Sollazzo[1,2], Elisa Monaca[1], Raffaele Sabbatella[1], Valeria Vetri[3], Rosa Passantino[4], Annalisa Pastore[5], Caterina Alfano[1]*

[1]Structural Biology and Biophysics Unit, Fondazione Ri.MED, Palermo 90133, Italy

[2] Department of Biological, Chemical and Pharmaceutical Sciences and Technologies (STEBICEF), University of Palermo, Palermo 90128, Italy

[3]Department of Physics and Chemistry - Emilio Segrè (DiFC), Università di Palermo, Palermo 90128, Italy

[4]Biophysics Institute, National Research Council, Palermo 90143, Italy

[5]European Synchrotron Radiation Facility, Ave des Martyrs, 38000 Grenoble, France

*To whom correspondence should be addressed

calfano@fondazionerimed.com

[ǂ] These authors contributed equally

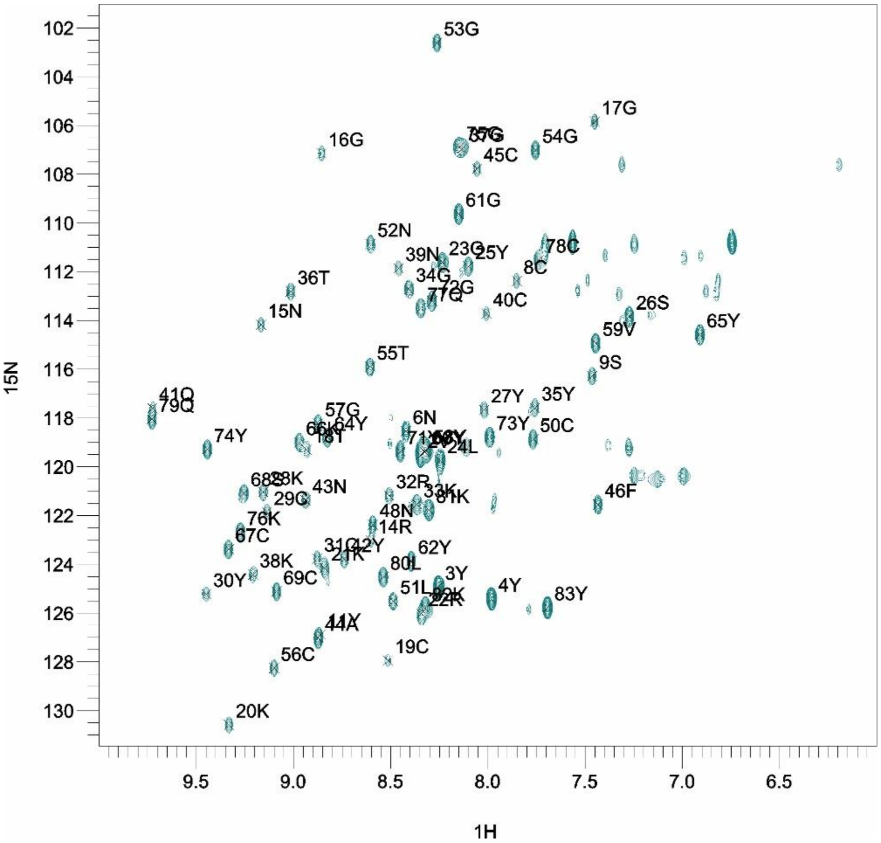

Supplementary Figure S1 - Assigned ^{15}N,^{1}H HSQC spectrum of recombinant Pvfp-5β. The cross peaks are labelled with single letter amino acid code followed by their position in the recombinant protein sequence. Wide dispersion of the peaks over a range of 9.7-6.7 ppm demonstrates that Pvfp-5β is well structured in its no DOPA-modified form.

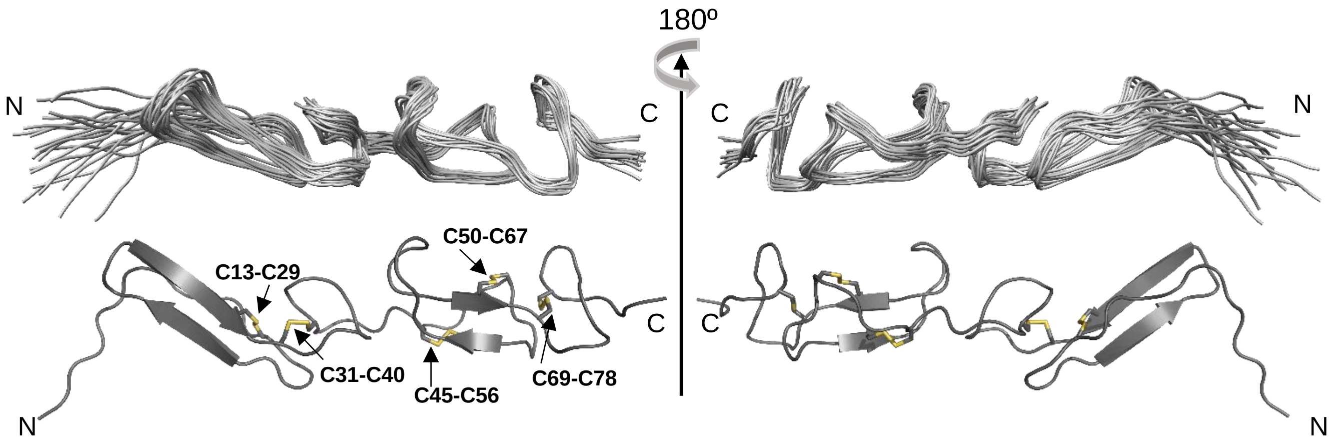

Supplementary Figure S2 - Pvfp-5b structure in solution obtained imposing only the five disulfide bridges found by MS. Top: superimposition of the 20 lowest energy structures. Bottom: cartoon representation of the structure with the lowest energy. Highlighted in yellow the five disulfide bridges.

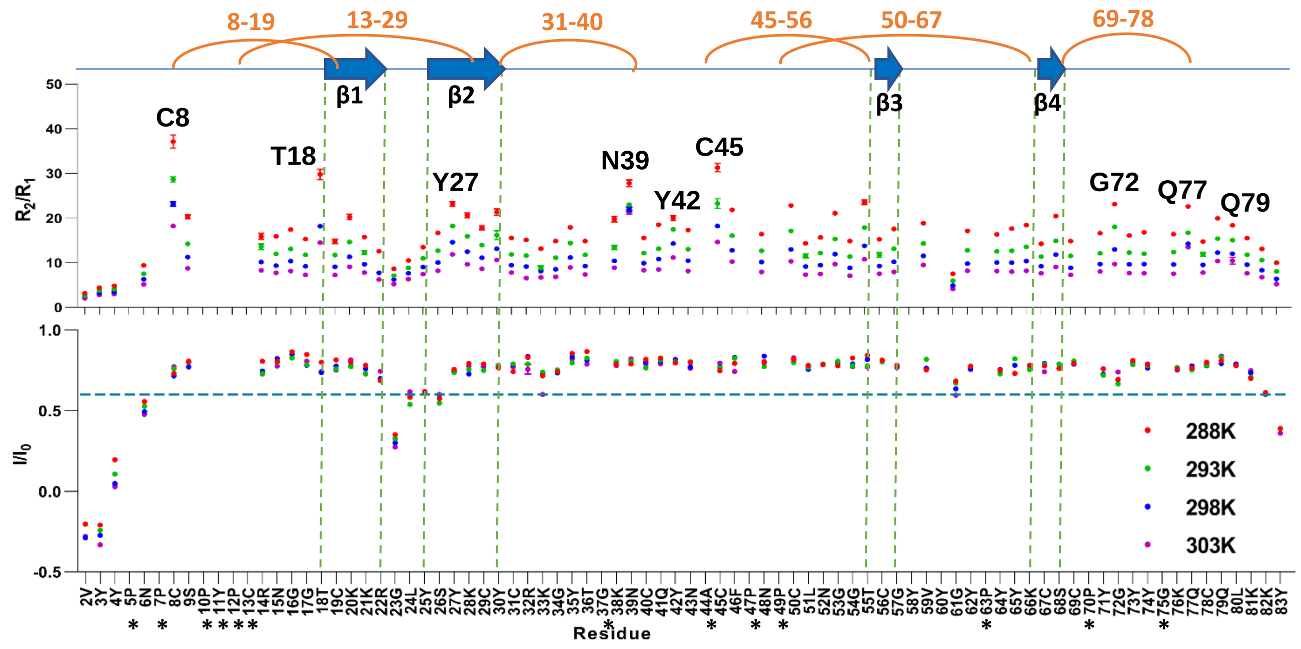

Supplementary Figure S3 - Relaxation measurements at different temperatures for Pvfp-5β. R_2/R_1 ratio (top) and hetNOE (bottom), recorded at 18.8T and different temperatures (288, 293, 298, and 303 K). β-strands are indicated as blue arrows at the top of the figure, while yellow curves indicate disulfide bonds. Black asterisks indicate proline residues or residues with peaks in overlap in the NMR experiments. In bold residues showing no-fast local motions within the μs-ms scale.

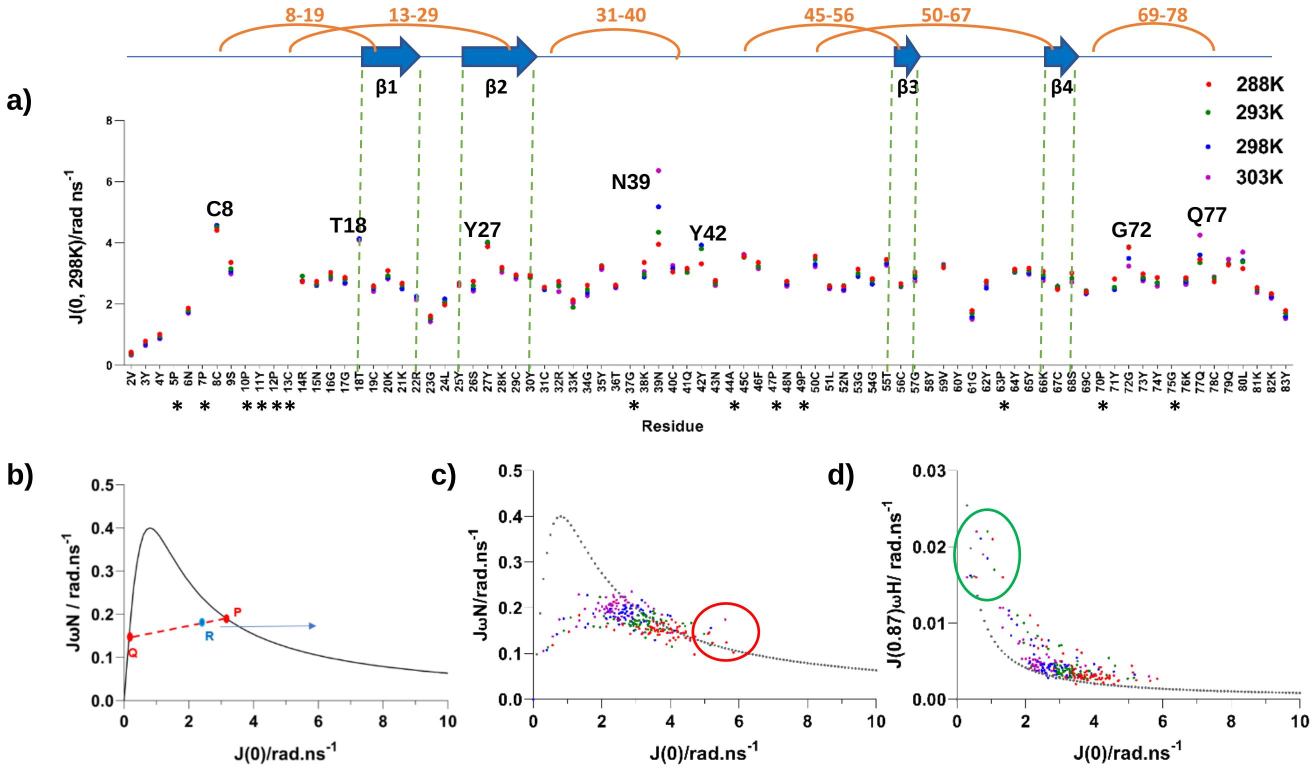

Supplementary Figure S4 - Reduced spectral density mapping for Pvfp-5β. A) Values of *J*(0, 298 K*)* obtained at 18.8 T as functions of the residue number. *J*(0*)* shows temperature dependence mostly given by the dependence of the overall tumbling to the viscosity-temperature ratio (η/T) for rigid residues. The effect of temperature was then compensated by multiplying *J*(0*)* with $T\eta_{ref}/\eta T_{ref}$, where T_{ref} is a chosen reference temperature (298 K in this study) and η_{ref} is viscosity at this temperature. The plot of the temperature-corrected *J(*0, 298K*)* facilitates the identification of deviations indicating conformational motions. B) Theoretical spectral density functions (solid curve) obtained using equation $J(\varpi) = \frac{J(0)}{1+6.25(\varpi J(0))^2}$. It represents all possible positions of points corresponding to the case of dynamics reduced to a single motion. Point P represents an N–H bond motion completely dominated by the overall isotropic tumbling. The *J(*0*)*-coordinate of P is thus equal to two-fifths of the overall rotational correlation time. In the same way, point Q represents an N–H bond completely dominated by a fast-internal motion. Real values of measured spectral density functions describing the pico- to nano-second dynamics should be found between these two limits, as represented by point R. In case of conformational motions on the micro- to milli-second time scale, *J*(0*)* increases in the direction of the blue arrow. C) Values of $J\omega N$ and D) $J(0.87)\omega H$ plotted in function of *J(0)* and compared to theoretical functions (grey dots). The distribution of the values indicates that the majority of the residues are clustered between Q and P, indicating they are rigid particles. Values shifted to the right (red circle in C)) are related to those residues experiencing motion in the μs-ms time scale (C8, T18, N39). Values in the green circle in D) are related to those residues moving faster than the global correlation time and are all belonging to the flexible N-terminal end of the protein (V2, Y3, Y4).

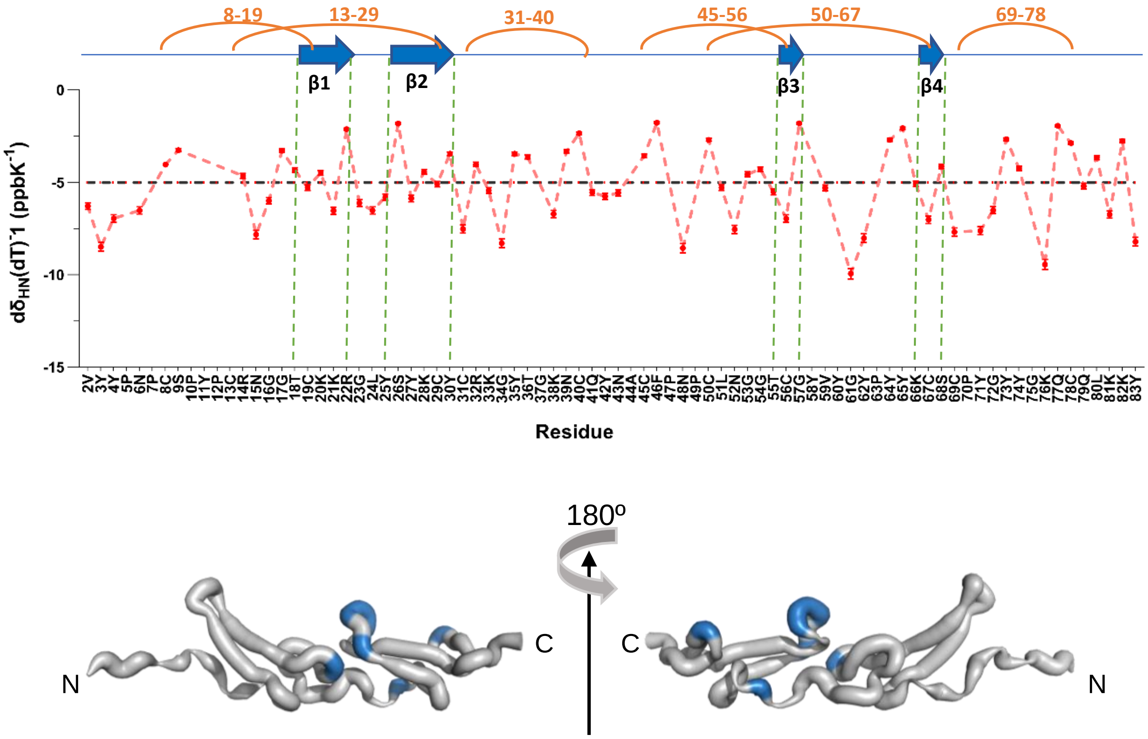

Supplementary Figure S5 - Thermal susceptibility of the HN amide protons of Pvfp-5β. Top: temperature dependence of the H_N chemical shift (dd_{HN}/dT*)* as a function of residue number. Dark stars indicate prolines or residues in overlap in the NMR experiments. Bottom: worm representations of Pvfp-5β structure. The thickness correlates to the dd_{HN}/dT, with the low-thermal susceptibility residues colored in grey (thin) and the high-thermal susceptibility ones in blue (thick). The thinner segments indicate residues with no information.

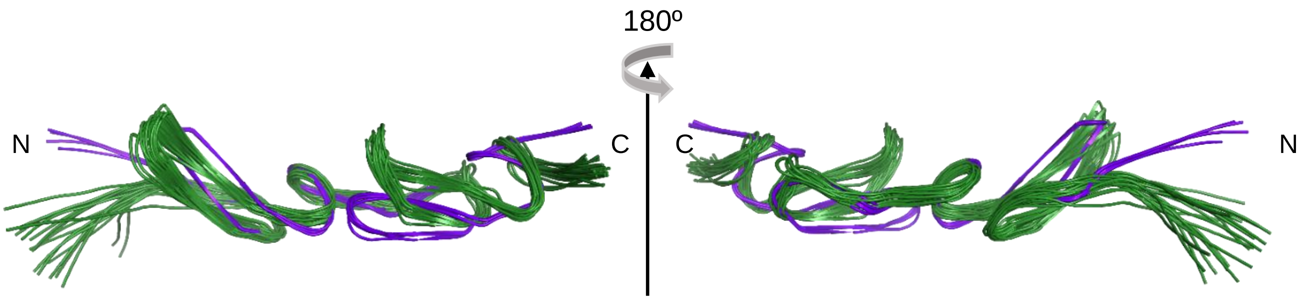

Supplementary Figure S6: Comparison Pvfp-5β experimental structure with AI models. In green the experimental structural ensemble of the 20 structures with lowest energy. In violet the five AI models obtained by RoseTTaFold.

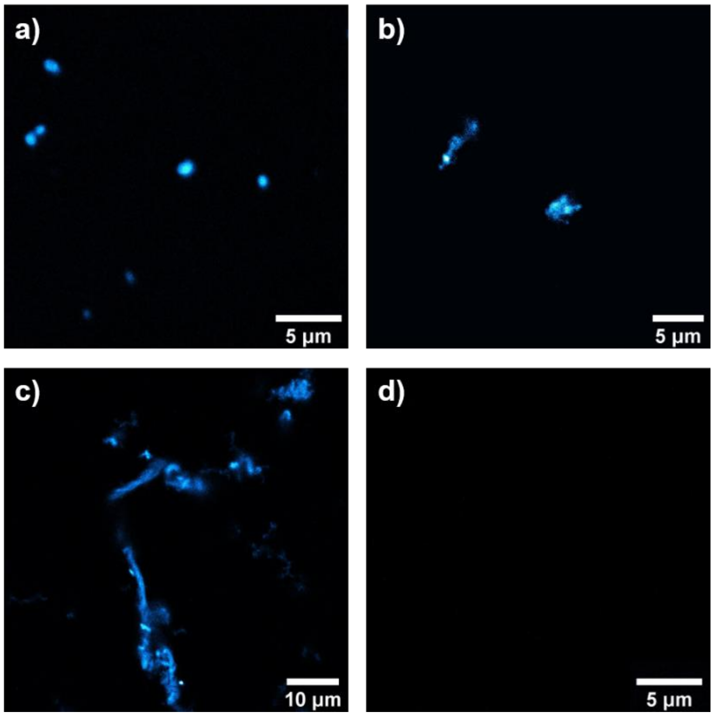

Supplementary Figure S7: Representative ThT fluorescence images of Pvfp5β coacervates and aggregates. 1024 x 1024 pixels laser scanning confocal microscopy images images of ThT stained samples show the progressive appearance of different species when Pvfp5β is suddenly diluted into 0.1M Tris-HCl pH 8, 1M NaCl: A) droplets of coacervation soon after the dilution in alkaline buffer; B) aggregates with different size at <30min from the dilution; C) fibrils at >1h from dilution; D) No ThT-positive species is detected in 20mM NaOAc pH 4.5.

Supplementary Table 1.
Oxidized chemical shifts for Cys

	Ca (ppm)	Cb (ppm)
C8	53.8	41.3
C13	52.5	38.1
C19	57.2	42.9
C29	54.4	42.6
C31	53.2	37.7
C40	54.9	36.2
C45	52.0	39.2
C50	53.2	38.2
C56	57.5	38.1
C67	54.2	42.3
C69	51.8	37.2
C78	54.8	35.3

Supplementary Table 2.
HADDOCK outputs for Pvfp-5β/Pvfp-5β dimer

Cluster ID number*	1
HADDOCK score	-89.1 +/- 7.6
Cluster size	177
RMSD from the overall lowest-energy structure	0.6 +/- 0.3
Van der Waals energy	-122.6 +/- 6.4
Electrostatic energy	-215.4 +/- 15.0
Desolvation energy	-39.9 +/- 4.1
Restraints violation energy	1164.9 +/- 70.52
Buried Surface Area	3247.5 +/- 64.7
Z-Score	-1.4

*Only clusters with negative Z-score are reported

Supplementary Table 3. Protein-Protein interactions in HADDOCK Pvfp-5β/Pvfp-5β dimer model

**π-cations**		**π-stacking**		**Hydrogen bond**	
Chain A	Chain B	Chain A	Chain B	Chain A	Chain B
Y64	K38	Y27	Y62	K76 (NH3)	P5 (C=O)
K38	Y64			Q77 (C=O)	N6 (NH2)
				K66 (NH3)	S26 (OH)
				G61 (C=O)	K28 (NH3)
				Y60 (C=O)	K28 (NH3)
				N43 (C=O)	N39 (NH2)
				Y35 (C=O)	N39 (NH2)
				N39 (NH2)	N43 (C=O)
				P10 (C=O)	N48 (NH2)
				K28 (NH3)	Y60 (C=O)
				K28 (NH3)	G61 (C=O)
				Q79 (C=O)	Y3 (OH)
