# Supplementary material for: Solution structure of recombinant Pvfp-5β reveals insights into mussel adhesion
Source: Commun Biol. 2022 Jul 25;5:739. doi: 10.1038/s42003-022-03699-w (PMC9314366; doi:10.1038/s42003-022-03699-w)
Supplement: Supplementary file 2 — Description of Additional Supplementary Files [file 42003_2022_3699_MOESM2_ESM.pdf]

Description of Additional Supplementary Files

File name: Supplementary Movie
Description: Coacervation of no-DOPA modified Pvfp-5β in alkaline condition.

File name: Supplementary Data
Description: The source data behind the graphs in the paper.
