# Supplementary material for: Solution structure of recombinant Pvfp-5β reveals insights into mussel adhesion
Source: Commun Biol. 2022 Jul 25;5:739. doi: 10.1038/s42003-022-03699-w (PMC9314366; doi:10.1038/s42003-022-03699-w)
Supplement: Supplementary file 4 — Supplementary Data [file 42003_2022_3699_MOESM4_ESM.pdf]

RELAXATION DATA AT 800 MHz AND 298 K											
Residue	R1 [rad/s]		R2 [rad/s]		NOE		R2/R1		J0	JwN	J0,87wH
2V	0.807622	0.000937	2.084179	0.003032	-0.28858	0.000101	2.580636	0.00675	0.39667	0.107916	0.016233
3Y	1.061219	0.001713	3.43033	0.006251	-0.273549	0.00014	3.232443	0.011108	0.691141	0.142172	0.021081
4Y	1.246129	0.002432	4.337517	0.009068	0.048386	0.000149	3.480794	0.01407	0.893472	0.176258	0.018497
5P											
6N	1.234004	0.003743	7.795515	0.026011	0.493392	0.000297	6.317253	0.04024	1.751587	0.187291	0.009751
7P											
8C	0.822142	0.0059	19.09597	0.306572	0.716389	0.000709	23.22709	0.539572	4.579996	0.129037	0.003637
9S	1.161	0.00482	13.11359	0.098407	0.771337	0.000465	11.29508	0.131655	3.070452	0.183702	0.004141
10P											
11Y											
12P											
13C											
14R	1.162146	0.009105	11.85369	0.153019	0.746574	0.000805	10.19983	0.211582	2.76091	0.183215	0.004594
15N	1.19266	0.00625	11.23684	0.088027	0.823838	0.000608	9.421664	0.12318	2.607023	0.190165	0.003277
16G	1.190564	0.005768	12.40292	0.100891	0.849348	0.000549	10.41769	0.135216	2.893783	0.190536	0.002798
17G	1.265777	0.005844	11.68324	0.087484	0.7807	0.000471	9.230096	0.111726	2.706552	0.200556	0.00433
18T	0.953496	0.006296	17.36199	0.23776	0.735599	0.00069	18.20877	0.369592	4.138242	0.150078	0.003932
19C	1.187433	0.008814	10.84908	0.130229	0.777205	0.00079	9.136579	0.177495	2.511743	0.188046	0.004127
20K	1.075339	0.007115	12.22858	0.13802	0.778735	0.000681	11.37183	0.203591	2.864277	0.170333	0.003711
21K	1.106311	0.005835	10.74298	0.074523	0.760067	0.000602	9.710625	0.11858	2.495705	0.174759	0.00414
22R	1.26392	0.00631	9.823425	0.057174	0.699522	0.000526	7.77219	0.084038	2.249034	0.19788	0.005924
23G	1.06767	0.003127	6.770319	0.020179	0.301524	0.000233	6.341212	0.037473	1.518709	0.15729	0.011632
24L	1.238382	0.003969	9.499103	0.031965	0.593692	0.000339	7.670576	0.050394	2.170767	0.190839	0.007848
25G	1.277917	0.005615	11.57581	0.060779	0.612361	0.000432	9.058338	0.087363	2.675466	0.197485	0.007727
26S	1.063166	0.003349	10.75416	0.046632	0.576505	0.000341	10.11522	0.075721	2.500918	0.163413	0.007023
27Y	1.156818	0.006652	16.9179	0.176451	0.744421	0.000644	14.62452	0.236626	4.003729	0.182317	0.004612
28K	1.07059	0.006818	13.38742	0.121017	0.727788	0.000652	12.50471	0.192675	3.148328	0.168314	0.004546
29C	1.110479	0.007149	12.4148	0.108431	0.786543	0.000684	11.17968	0.169618	2.905661	0.1761	0.003697
30Y	0.947769	0.007207	12.47131	0.165005	0.773165	0.000724	13.15859	0.274164	2.939813	0.150003	0.003353
31C	1.123204	0.005121	10.65693	0.076404	0.782608	0.000527	9.487968	0.111279	2.47278	0.178016	0.003809
32R	1.228973	0.005957	11.29	0.070582	0.836283	0.000534	9.186533	0.101959	2.615757	0.19631	0.003138
33K	1.127327	0.003938	9.243727	0.03495	0.72505	0.000378	8.199684	0.059647	2.124609	0.177163	0.004835
34G	1.196956	0.005257	10.28793	0.056521	0.735312	0.000476	8.595077	0.084969	2.372132	0.18839	0.004942
35Y	1.218401	0.005425	13.68318	0.107618	0.834796	0.000538	11.23044	0.138328	3.204092	0.194579	0.00314
36T	1.195836	0.004731	11.11859	0.064111	0.813118	0.000452	9.297757	0.090393	2.577427	0.190374	0.003486
37G											
38K	1.183001	0.007471	12.39527	0.123249	0.800769	0.000676	10.47781	0.170356	2.89201	0.187992	0.003676
39N	0.983293	0.00638	21.63636	0.364702	0.789414	0.000728	22.00398	0.513662	5.183805	0.155997	0.00323
40C	1.365754	0.008141	13.62593	0.157385	0.801141	0.000659	9.976854	0.174704	3.170929	0.217045	0.004236
41Q	1.237213	0.009108	13.46346	0.192695	0.806545	0.000704	10.88208	0.23586	3.147319	0.196772	0.003733
42Y	1.157386	0.005264	16.60318	0.1871	0.812798	0.000534	14.34542	0.226907	3.927635	0.184244	0.00338
43N	1.098535	0.004705	11.55773	0.076846	0.771362	0.000475	10.52104	0.115011	2.696669	0.173819	0.003918
44A											
45C	0.816845	0.004292	14.91538	0.166959	0.769434	0.000517	18.25975	0.300345	3.555016	0.129211	0.002938
46F	1.069538	0.003174	13.73356	0.077407	0.830314	0.000355	12.84064	0.110482	3.235007	0.170695	0.002831
47P											
48N	1.118441	0.00401	11.39733	0.067496	0.838413	0.000455	10.19037	0.096886	2.655951	0.17871	0.002819
49P											
50C	1.072913	0.003022	13.98435	0.088483	0.814392	0.000361	13.03401	0.119186	3.295848	0.170837	0.003106
51L	1.197903	0.005902	10.99816	0.096129	0.756414	0.000483	9.181175	0.125482	2.54664	0.189126	0.004551
52N	1.129302	0.004166	10.7191	0.059209	0.788018	0.000396	9.4918	0.087449	2.487355	0.179124	0.003734
53G	1.037074	0.003254	12.40024	0.085024	0.798764	0.000347	11.95695	0.119499	2.911548	0.164754	0.003255
54G	1.289827	0.00402	11.49026	0.074155	0.789817	0.00033	8.908371	0.085257	2.656361	0.204639	0.004229
55T	1.023052	0.00381	14.13825	0.097715	0.81852	0.000441	13.81967	0.146985	3.339916	0.162996	0.002896
56C	1.198377	0.00531	11.13234	0.092071	0.810069	0.000494	9.28952	0.117993	2.58046	0.190694	0.00355
57G	1.201012	0.004251	12.34298	0.082712	0.776275	0.000403	10.27715	0.105244	2.876462	0.190171	0.004191
58Y											
59V	1.188766	0.004444	13.74273	0.080296	0.763582	0.000409	11.5605	0.110762	3.221147	0.187881	0.004384
60Y											
61G	1.468312	0.003037	7.214774	0.016302	0.635091	0.000226	4.913653	0.021267	1.581724	0.227683	0.008357
62Y	1.101145	0.002965	10.85928	0.048857	0.756684	0.000303	9.861814	0.070925	2.524782	0.173857	0.004179

63P											
64Y	1.301713	0.004955	13.12959	0.08201	0.754935	0.000429	10.0864	0.101396	3.05632	0.205471	0.004976
65Y	1.290748	0.003727	12.98375	0.068443	0.782355	0.000332	10.05909	0.082072	3.022458	0.204562	0.004382
66K	1.18271	0.004929	12.32542	0.080777	0.7775	0.000482	10.42133	0.111727	2.874485	0.187306	0.004105
67C	1.187969	0.004504	11.01625	0.061359	0.793254	0.000449	9.273187	0.086811	2.552958	0.188574	0.003831
68S	1.020088	0.004277	12.11657	0.078037	0.762992	0.000414	11.87797	0.126304	2.843518	0.161208	0.003771
69C	1.140921	0.00357	10.13343	0.052235	0.788755	0.000347	8.881802	0.073572	2.342253	0.180986	0.003759
70P											
71Y	1.094394	0.002682	10.67231	0.040467	0.725666	0.000278	9.751803	0.060877	2.479267	0.172003	0.004683
72G	1.137263	0.00356	14.84389	0.089112	0.692647	0.000338	13.05229	0.119215	3.496559	0.177869	0.005452
73Y	1.253178	0.003403	12.18525	0.059606	0.793216	0.000347	9.72348	0.073971	2.831517	0.198924	0.004042
74Y	1.186968	0.003729	11.56676	0.061797	0.765031	0.000351	9.744797	0.082677	2.687625	0.187637	0.00435
75G											
76K	1.21811	0.004518	11.75642	0.059599	0.753893	0.000411	9.651365	0.084724	2.73002	0.192245	0.004676
77Q	1.066196	0.003899	15.25498	0.114939	0.777708	0.000425	14.30786	0.160124	3.607797	0.168859	0.003697
78C	1.293463	0.00543	12.32359	0.088914	0.778828	0.000442	9.527599	0.108739	2.86011	0.204886	0.004462
79Q	1.139149	0.004173	14.0191	0.099906	0.791073	0.00044	12.30665	0.132786	3.29567	0.180767	0.003712
80L	1.206154	0.004531	14.55431	0.11878	0.789153	0.000417	12.06672	0.14381	3.418203	0.191345	0.003967
81K	1.108028	0.003205	10.62179	0.036841	0.735384	0.00031	9.586214	0.060981	2.465294	0.174396	0.004573
82K	1.16346	0.003291	9.766153	0.03611	0.613121	0.000281	8.394063	0.054782	2.246257	0.179818	0.007021
83Y	1.106616	0.001917	7.112101	0.012581	0.390051	0.000155	6.42689	0.022502	1.598824	0.165302	0.010528

RELAXATION DATA AT 600 MHz AND 298 K											
**Residue**	**R1 [rad/s]**		**R2 [rad/s]**		**NOE**		**R2/R1**		**J(0)**	**JwN**	**J(0.87)Wh**
2V	0.877068	0.000606	2.180355	0.00259	-0.627797	0.002871	2.485959	0.004672	0.482386	0.131048	0.022269
3Y	1.180736	0.001109	3.13561	0.00343	-0.467674	0.003293	2.655641	0.005399	0.711152	0.181637	0.02703
4Y	1.400682	0.001583	7.220551	0.00784	-0.174166	0.002649	5.155024	0.011424	1.871338	0.226812	0.025653
5P											
6N	1.543107	0.003068	7.212827	0.022695	0.398559	0.005904	4.674223	0.024	1.86104	0.274254	0.014476
7P											
8C	1.175232	0.004739	15.53228	0.124406	0.676366	0.014441	13.21635	0.159146	4.349308	0.217878	0.005933
9S	1.58966	0.004371	11.95352	0.074433	0.730883	0.009655	7.519546	0.067501	3.244991	0.2971	0.006673
10P											
11Y											
12P											
13C											
14R	1.574133	0.007831	10.22646	0.12421	0.703221	0.015668	6.496565	0.111226	2.743109	0.292997	0.007287
15N	1.666176	0.005901	10.70034	0.052351	0.769872	0.013091	6.422099	0.054163	2.869298	0.313193	0.005981
16G	1.59897	0.004501	12.2774	0.054883	0.795235	0.011074	7.678321	0.055936	3.339814	0.301679	0.005107
17G	1.733357	0.005161	11.03823	0.048772	0.771161	0.010773	6.368121	0.047098	2.957762	0.325883	0.006187
18T	1.370212	0.00599	14.58765	0.103582	0.707364	0.015388	10.64627	0.122133	4.045267	0.255198	0.006254
19C	1.601998	0.007425	9.891893	0.064747	0.738021	0.015152	6.17472	0.069035	2.64236	0.299722	0.006546
20K	1.471779	0.005857	15.82957	0.114452	0.725276	0.014615	10.7554	0.120565	4.392315	0.274841	0.006307
21K	1.520767	0.00483	9.666114	0.034008	0.689548	0.011092	6.356079	0.042548	2.587457	0.282491	0.007364
22R	1.720417	0.00535	9.475154	0.038181	0.658434	0.010855	5.507475	0.03932	2.500646	0.3181	0.009166
23G	1.28117	0.002322	5.919498	0.011933	0.227793	0.004399	4.620385	0.017687	1.521147	0.221665	0.015432
24L	1.543374	0.003539	8.422251	0.022125	0.584223	0.008148	5.457037	0.026847	2.21853	0.282206	0.010009
25G	1.659858	0.004476	10.23414	0.055383	0.532327	0.00829	6.165672	0.049994	2.727364	0.301129	0.012108
26S	1.400665	0.003492	9.328347	0.027165	0.531664	0.007824	6.659941	0.035998	2.50325	0.254081	0.010232
27Y	1.547505	0.005262	14.01674	0.072733	0.713037	0.012326	9.057637	0.077797	3.852175	0.28846	0.006927
28K	1.479564	0.005934	11.15612	0.091197	0.723389	0.013644	7.540141	0.091878	3.028903	0.276218	0.006384
29C	1.5413	0.006103	10.27614	0.056289	0.729662	0.014351	6.667194	0.062922	2.763265	0.28801	0.006499
30Y	1.354478	0.006197	12.35208	0.092478	0.768731	0.016635	9.119442	0.109999	3.397529	0.25456	0.004886
31C	1.564619	0.004563	9.537262	0.047971	0.708246	0.010593	6.09558	0.048437	2.543787	0.291443	0.00712
32R	1.694291	0.005925	10.05888	0.043574	0.772554	0.012016	5.936923	0.046481	2.678192	0.318603	0.006011
33K	1.544119	0.003568	6.95849	0.021634	0.632856	0.007854	4.506448	0.024425	1.793163	0.284413	0.008843
34G	1.638977	0.0051	9.663353	0.038067	0.646638	0.010539	5.895968	0.041571	2.567525	0.302509	0.009034
35Y	1.612543	0.005016	11.97037	0.063063	0.755829	0.012395	7.423289	0.062198	3.247116	0.302487	0.006142
36T	1.661698	0.004203	9.674923	0.046236	0.737433	0.010125	5.822312	0.042553	2.570107	0.310864	0.006806
37G											
38K	1.602708	0.00637	10.85199	0.056426	0.772375	0.013889	6.771036	0.062117	2.92308	0.301373	0.00569
39N	1.408254	0.006353	17.45471	0.17643	0.744462	0.016066	12.39457	0.181195	4.876052	0.263724	0.005613
40C	1.866607	0.007435	12.46608	0.086136	0.782787	0.015088	6.67847	0.072747	3.354456	0.351533	0.006324

41Q	1.670411	0.006237	12.02933	0.10127	0.758559	0.013124	7.201419	0.087516	3.255727	0.313468	0.006291
42Y	1.58604	0.004598	13.05421	0.093994	0.777928	0.010628	8.230695	0.083122	3.56761	0.298482	0.005494
43N	1.538788	0.004111	9.820162	0.039439	0.763909	0.010584	6.38175	0.042681	2.631664	0.288995	0.005667
44A											
45C	1.209377	0.003798	14.48131	0.125644	0.734044	0.010938	11.97419	0.141494	4.039785	0.226133	0.005017
46F	1.457805	0.003008	10.89933	0.060036	0.739201	0.0073	7.476531	0.056609	2.958065	0.272792	0.00593
47P											
48N	1.540844	0.003771	9.576119	0.037026	0.73311	0.008537	6.214853	0.039239	2.55938	0.288072	0.006414
49P											
50C	1.466385	0.002692	12.20707	0.0442	0.741783	0.007834	8.324604	0.045427	3.337677	0.274502	0.005906
51L	1.632872	0.004817	12.73833	0.090072	0.756909	0.010852	7.801186	0.078176	3.467941	0.306349	0.006191
52N	1.554113	0.004003	9.677402	0.038586	0.743775	0.008853	6.226961	0.040867	2.587199	0.29101	0.006211
53G	1.407819	0.002599	11.12089	0.044185	0.762187	0.007541	7.899375	0.045967	3.0304	0.264331	0.005222
54G	1.745438	0.002843	12.57331	0.057121	0.738927	0.006608	7.203528	0.044458	3.402394	0.326602	0.007108
55T	1.430483	0.003582	12.6831	0.054437	0.769135	0.009211	8.866307	0.060256	3.482598	0.26886	0.005151
56C	1.62728	0.004397	10.16066	0.055073	0.788899	0.011041	6.24395	0.050715	2.718461	0.306736	0.005358
57G	1.676146	0.004183	11.09688	0.04728	0.73921	0.00924	6.620472	0.044728	2.982473	0.313649	0.006818
58Y											
59V	1.643981	0.004652	12.3123	0.048082	0.735838	0.009264	7.489322	0.050441	3.341535	0.307477	0.006774
60Y											
61G	1.697314	0.002229	6.844619	0.009962	0.532303	0.004423	4.032619	0.011165	1.733618	0.307923	0.012382
62Y	1.496123	0.002423	9.620025	0.021378	0.725611	0.006742	6.429968	0.024704	2.578708	0.279401	0.006403
63P											
64Y	1.796928	0.004697	11.46544	0.053827	0.70933	0.008592	6.380576	0.046634	3.070839	0.33477	0.008147
65Y	1.761341	0.003538	11.39739	0.038488	0.740457	0.007343	6.470862	0.03485	3.057298	0.329652	0.007131
66K	1.623782	0.00469	11.2625	0.057136	0.735366	0.011303	6.935973	0.055222	3.038554	0.303678	0.006703
67C	1.586548	0.004017	9.532526	0.030782	0.72234	0.009097	6.008345	0.034616	2.539494	0.296145	0.006871
68S	1.44655	0.004094	10.3564	0.046654	0.733058	0.009835	7.15938	0.052516	2.801019	0.270441	0.006023
69C	1.530826	0.00276	9.977397	0.028098	0.738682	0.007622	6.517658	0.030105	2.677957	0.286434	0.00624
70P											
71Y	1.519215	0.002542	9.533669	0.022377	0.666611	0.005872	6.275393	0.02523	2.548466	0.281241	0.0079
72G	1.546129	0.003242	12.20177	0.04143	0.651157	0.007488	7.891815	0.043343	3.321662	0.285564	0.008413
73Y	1.650982	0.003005	11.31583	0.051516	0.701999	0.006747	6.853997	0.043677	3.048999	0.307246	0.007674
74Y	1.645829	0.003454	10.94169	0.04925	0.742523	0.007759	6.648128	0.043876	2.942008	0.308127	0.00661
75G											
76K	1.632395	0.004183	10.26314	0.032752	0.697448	0.009019	6.287168	0.036175	2.744835	0.303582	0.007704
77Q	1.495163	0.003768	13.60793	0.076474	0.734678	0.009388	9.101305	0.074083	3.741565	0.279596	0.006188
78C	1.747137	0.004304	11.1349	0.070788	0.744836	0.009486	6.373227	0.056216	2.98308	0.327205	0.006954
79Q	1.587401	0.003974	12.49558	0.057	0.74645	0.009161	7.871721	0.055615	3.403719	0.29736	0.006278
80L	1.638796	0.003927	11.73128	0.068323	0.75944	0.00893	7.158471	0.058845	3.173598	0.307575	0.006149
81K	1.519026	0.002743	10.07371	0.036071	0.676296	0.006588	6.631693	0.03572	2.706235	0.281612	0.00767
82K	1.551273	0.002695	11.55303	0.031296	0.570378	0.005721	7.447446	0.033113	3.129561	0.283058	0.010395
83Y	1.377093	0.001426	8.76235	0.015199	0.27	0.002542	6.362931	0.017627	2.335512	0.239865	0.01568
